# Reconciling diverse mammalian pigmentation patterns with a fundamental mathematical model

**DOI:** 10.1038/ncomms10288

**Published:** 2016-01-06

**Authors:** Richard L. Mort, Robert J. H. Ross, Kirsten J. Hainey, Olivia J. Harrison, Margaret A. Keighren, Gabriel Landini, Ruth E. Baker, Kevin J. Painter, Ian J. Jackson, Christian A. Yates

**Affiliations:** 1 MRC Human Genetics Unit, MRC IGMM, Western General Hospital, University of Edinburgh, Edinburgh EH4 2XU, UK; 2 Wolfson Centre for Mathematical Biology, University of Oxford, Andrew Wiles Building, Woodstock Road, Oxford OX2 6GG, UK; 3 Oral Pathology Unit, School of Dentistry, College of Medical and Dental Sciences, University of Birmingham, St Chad's, Queensway, Birmingham B4 6NN, UK; 4 Department of Mathematics and Maxwell Institute for Mathematical Sciences, Heriot-Watt University, Edinburgh EH14 4AS, UK; 5 Roslin Institute, University of Edinburgh, Roslin EH25 9RG, UK; 6 Centre for Mathematical Biology, Department of Mathematical Sciences, University of Bath, Claverton Down, Bath BA2 7AY, UK

## Abstract

Bands of colour extending laterally from the dorsal to ventral trunk are a common feature of mouse chimeras. These stripes were originally taken as evidence of the directed dorsoventral migration of melanoblasts (the embryonic precursors of melanocytes) as they colonize the developing skin. Depigmented ‘belly spots' in mice with mutations in the receptor tyrosine kinase Kit are thought to represent a failure of this colonization, either due to impaired migration or proliferation. Tracing of single melanoblast clones, however, has revealed a diffuse distribution with high levels of axial mixing—hard to reconcile with directed migration. Here we construct an agent-based stochastic model calibrated by experimental measurements to investigate the formation of diffuse clones, chimeric stripes and belly spots. Our observations indicate that melanoblast colonization likely proceeds through a process of undirected migration, proliferation and tissue expansion, and that reduced proliferation is the cause of the belly spots in Kit mutants.

## Abstract (web summary)

How embryonic melanoblast behaviour influences adult pigmentation patterns and causes patterning defects is unclear. Here, Mort *et al*. construct a stochastic model parameterised experimentally to show that melanoblast migration is undirected and that reduced proliferation causes patterning defects.

## Graphical abstract

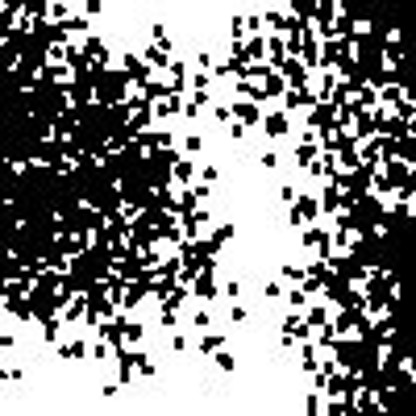

Melanoblasts, the embryonic precursors of the pigment producing melanocytes of skin and hair, colonize the developing epidermis during development. Mice heterozygous for mutations of the receptor tyrosine kinase Kit present with a depigmented ventral belly spot long thought to represent a failure of either melanoblast proliferation or migration^1^. Melanoblasts are specified at embryonic day 9 (E9) in the pre-migratory neural crest^2^ by downregulation of the transcription factors Foxd3 and Sox2 and upregulation of the master transcription factor Mitf^3^,^4^. At E10.5 melanoblasts delaminate from the neural crest, upregulate the melanoblast-specific genes *Pmel* and *Dct*^5^,^6^, and accumulate in a region known as the migration staging area (MSA)^1^,^7^,^8^. Melanoblast survival in the skin is dependent on signalling between Kit (expressed in melanoblasts) and its ligand Kitl (expressed by dermal fibroblasts and keratinocytes), however initial delamination does not require Kit signalling^1^,^9^. At E10.5 melanoblasts begin to leave the MSA in a Kitl- and endothelin 3 (Edn3)-dependent manner and embark on a dorsolateral migratory pathway in the dermis between the developing somites and the ectoderm^1^,^7^,^8^,^10^. At E12.5 melanoblasts move from the dermis to the epidermis, upregulate E-cadherin, lose their dependence on Edn3 and continue to migrate and proliferate^11^,^12^,^13^. A dermal population also persists whose size remains constant (and so proportionally decreases in relation to the epidermal population)^14^. Epidermal colonization is complete by around E15.5, after which melanoblasts downregulate E-cadherin and begin to localize to the developing hair follicles^11^,^15^,^16^. Luciani *et al*.^14^ measured melanoblast doubling times in the dermis and epidermis, and used a mathematical model to estimate the number of melanoblast progenitors specified in the pre-migratory neural crest^14^.

Dorsolateral migration was first demonstrated by the capacity of grafted mouse skin to produce melanin in chick coelom^17^,^18^. Later, this early work was confirmed in chimeric mice generated by the aggregation of albino and pigmented cleavage-stage embryos^19^. In this work, Mintz described a ‘standard pattern' of 17 successive bands/stripes extending dorsoventrally. Although the interpretation of the number of stripes was later criticized^20^,^21^, the acceptance of the stripes as representing directed dorsolateral migration remained. However, using a *Dct::laacZ* transgene to label individual *lacZ* revertant melanoblast clones, Wilkie *et al*.^22^ observed a surprising degree of mixing at the axial level. The patterns observed were hard to reconcile with directed migration and suggested that different cell dispersal mechanisms may act in the head and trunk^22^.

Experimental measurements and mathematical modelling have formed the basis of a number of studies on neural crest colonization. The failure to colonize the embryonic intestine by neural crest precursors in Hirschsprung's disease has been extensively studied^23^,^24^,^25^,^26^,^27^, as has the behaviour of chick cranial neural crest populations^28^. Both continuous partial differential equation population-level models^23^ and discrete lattice-based individual-level models^29^ have been employed to model Hirschsprung's disease. Zhang *et al*.^29^ demonstrated, using a lattice-based model, that colonization of the intestine is more sensitive to changes in proliferation rate than in cell migration^23^,^29^. Stochastic effects, proposed to underlie incomplete penetrance in some neurocristopathies^30^, have also been investigated using individual-level modelling approaches. In the present study we use experimental observations to parameterize an agent-based stochastic model that allows us to explore colonization of the trunk epidermis by migrating melanoblasts. We conclude that a simple mechanism of undirected migration, proliferation and tissue expansion can reconcile the patterns described above. Furthermore, Kit mutant melanoblasts migrate normally but have a proliferation defect that results in a depigmented ventral belly spot.

## Results

### Dermal and epidermal melanoblast migration is undirected

**Figure 1:**
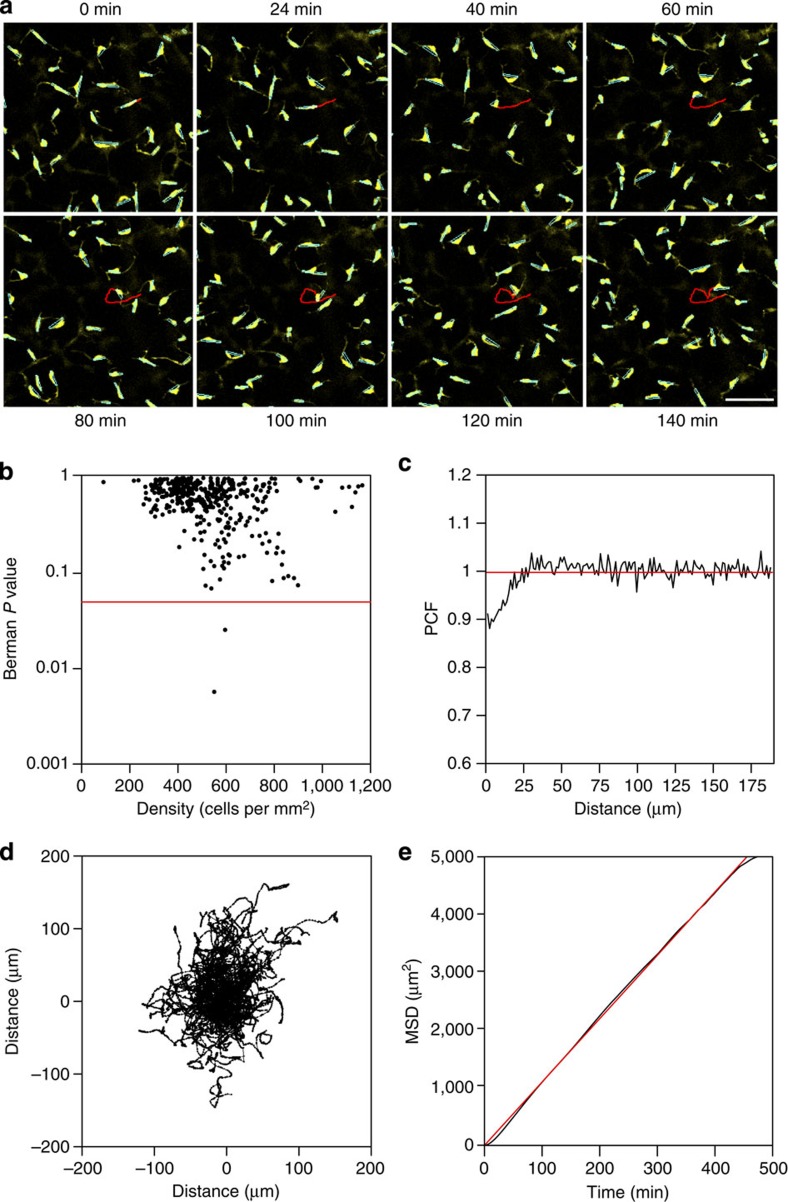
Melanoblast migration is undirected and not driven by repulsion. (**a**) A time-lapse sequence of melanoblasts migrating in *ex vivo* culture of E14.5 skin. The Feret's diameter of each cell body is indicated in cyan. The path of a single migrating cell is indicated in red. Melanoblasts migrate along their Feret's diameter—the longest distance between any two points along a given boundary. The distribution of the Feret's angles in the time-lapse sequences invariably conformed to a uniform distribution (Kolmogorov–Smirnov Test *P*>0.05 in all cases, *n*=414 independent tests) implying that melanoblast migration is undirected. (**b**) An analysis of the spacing of melanoblasts in the time-lapse sequences at E14.5. A range of cell densities was observed at the start of each time-lapse (*t*=0) between 242 and 780 cells per mm^2^ (mean±95% CI=448.52±57.46 cells per mm^2^, *n*=20). Independent spatial patterns were generated by sampling each time-lapse at 40-min intervals. Each spatial pattern (*n*=414 patterns) representing the *x*, *y* position of the centre of mass of each cell was tested for complete spatial randomness (CSR) using a Berman's test for a point process model. In the majority of examples the pattern conforms to CSR (*n*=412 out of 414 independent tests). The 0.05 significance line is indicated in red, the Berman's *P* value is plotted against the cell density at *t*=0. (**c**) Average PCF for melanoblast spatial organization at E14.5 for data in **b**. The red line indicates complete spatial randomness at all distances. Melanoblast spacing conforms to CSR at distances over ∼28 μm. (**d**) All tracks of a time-lapse sequence plotted from a zero origin showing a homogeneous population spread. (**e**) Plot of the MSD of the tracks panel 1d against time. A straight line through the origin (red) can be fitted to the data (black) indicating that the population is diffusing. The slope of the line is used to derive the diffusion coefficient (*D*). PCF, pairwise correlation function. Scale bar in **a**, 50 μm.

To better understand the behaviour of melanoblasts during their colonization of the developing embryo, and to understand how this behaviour may result in the patterns described above (Supplementary Fig. 1a,b), we performed *ex vivo* live imaging of whole mouse embryos at E11.5 and E12.5 and of mouse embryonic skin at E14.5 (refs 31, 32). At E14.5 we used a *Tyr::Cre* transgene expressing Cre-recombinase under the control of the tyrosinase promoter with *R26R-EYFP* to label melanoblasts with EYFP. At earlier stages, where *Tyr::Cre* activity in other neural crest lineages made imaging of melanoblasts difficult, we used a *Pmel::CreERT2* knock-in line combined with *R26R-EYFP* (Methods). In all time-lapse sequences between E11.5 (Supplementary Movies 1 and 2) and E15.5 melanoblasts migrated constantly, only pausing when they briefly pass through M-phase (Supplementary Movie 3). They are polarized and move along their longest axis (or Feret's diameter—see Fig. 1a and Supplementary Movie 4). The Feret's angle is therefore a good proxy for the direction of instantaneous migration. The distribution of these angles in our time-lapse sequences is uniform, consistent with completely random melanoblast orientation and undirected migration. To confirm that this was not an artefact of the *ex vivo* system we stained *Dct::lacZ* embryos expressing β-galactosidase in the melanoblast lineage^6^ and observed the same random distribution of melanoblast angles at time points between E11.5 and E15.5 (Supplementary Table 1). There is thus no directionality associated with melanoblast migration in developing dermis at E11.5–E12.5 or epidermis at E13.5–E15.5. We therefore assume this is also the case at E10.5 when cell numbers are too low to perform a rigorous analysis.

### Melanoblast dispersal is not mediated by repulsion

Time-lapse sequences were sampled from E14.5 embryos in the mid-trunk region at a range of dorsoventral positions resulting in a range of melanoblast densities. To investigate whether any mechanism of mutual repulsion between adjacent melanoblasts influenced their position, we tested whether the spacing of melanoblasts conformed to complete spatial randomness (CSR)^33^. There was no association between melanoblast density and the *P* value of a Berman's test comparing each pattern with CSR, and 99.5% of examples did not deviate from CSR (Fig. 1b). We reasoned that there should still be an effect over very short distances due to exclusion at the scale of single cells and so examined the pair-correlation function (PCF)^34^,^35^,^36^, a summary statistic that provides a measure of spatial patterning on different scales (Methods). This showed a non-random spatial segregation of melanoblasts below around 28 μm (Fig. 1c), consistent with volume exclusion at the scale of an individual cell. Above this distance the spacing of cells conformed to CSR. The melanoblast distribution at E14.5 (before hair follicle localization) is therefore spatially random, and repulsion between melanoblasts does not contribute to their dispersal.

### Melanoblast migration/proliferation are density dependent

**Figure 2:**
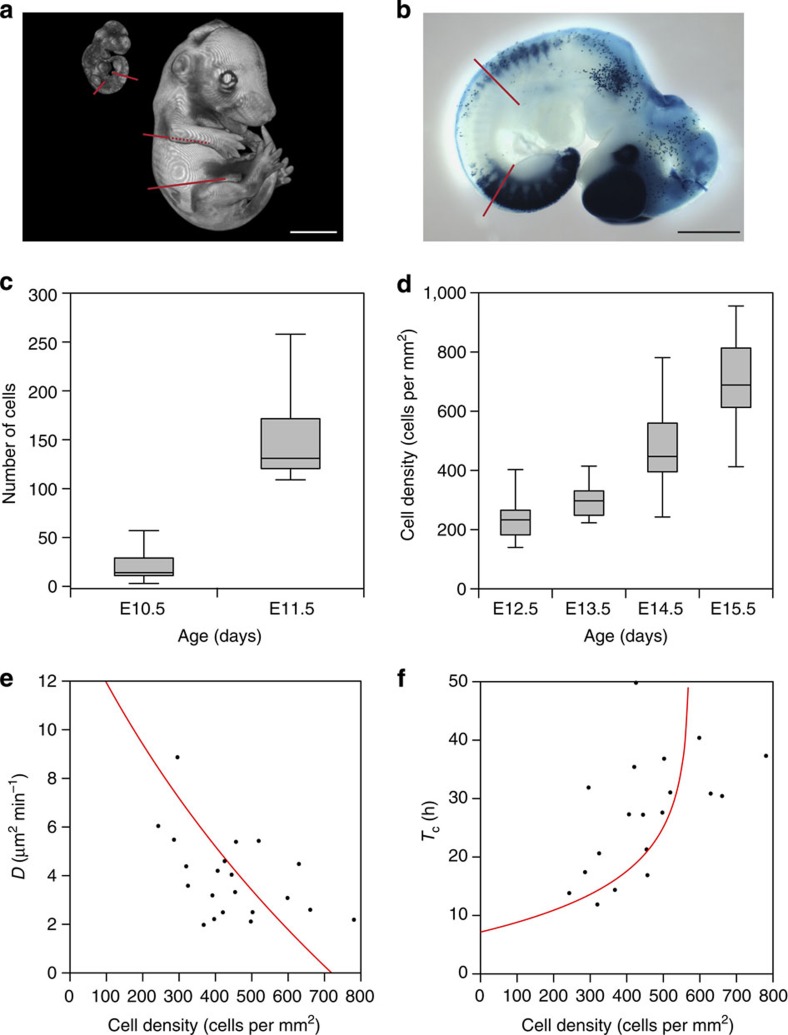
Key biological parameters. (**a**) For our experimental measurements we only considered the trunk region between the limb buds indicated by the coloured lines on the optical projection tomography (OPT) models at E10.5 (left) and E15.5 (right). This was defined as the axial width. (**b**) We used X-Gal-stained *Dct::lacZ* embryos to analyse cell numbers between E10.5 and E11.5. (**c**) Melanoblast numbers increase around ∼6-fold between E10.5 (*n*=25) and E11.5 (*n*=11) accompanied by an increase in axial width of ∼200 μm and in dorsoventral length of ∼1,000 μm. (**d**) Melanoblast mid-trunk density at E12.5 (*n*=8), E13.5 (*n*=8), E14.5 (*n*=32) and E15.5 (*n*=7) accompanied by linear increases in axial width of ∼900 μm and dorsoventral length of ∼3,200 μm (between E12.5 and E15.5). (**e**) The diffusion coefficient (*D*) was defined for each time-lapse experiment (*n*=20 E14.5 wild-type samples) and the values plotted against the initial density for each time-lapse. Pearson's product-moment correlation indicates a significant negative correlation between diffusion (*D*) and density (*r*=−0.49, degrees of freedom (df)=18, *P*=0.026). The functional form of the relationship between the diffusion coefficient and the cell density is recapitulated as an emergent property of the model (red line). (**f**) Analysis of cell cycle time (*T*_c_) in E14.5 time-lapse sequences (*n*=19 wild-type samples). Pearson's product-moment correlation indicates a significant positive association between cell cycle time (*T*_c_) and density (*r*=0.55, df=17, *P*=0.016). The functional form of the relationship between cell cycle time (*T*_c_) and cell density described is as an emergent property of the stochastic model (red line). Scale bars in **a**, 1,500 μm; in **b**, 1,000 μm. Whiskers in bold=maximum and minimum of all data. The boxes enclose the 2nd and 3rd quartiles.

We examined the population spread of melanoblasts in our E14.5 time-lapse sequences and calculated its mean squared displacement (MSD). We found MSD to be directly proportional to time, indicating that diffusion is an appropriate mathematical description of melanoblast migration and consequently that the melanoblast population is not migrating with a preferred direction (Fig. 1d,e). Similar results were observed at E13.5 and E15.5. Owing to the small number of melanoblasts at E11.5 we were not able to perform a diffusion analysis but their behaviour appeared qualitatively similar to E14.5 melanoblasts (compare Supplementary Movies 1 and 2 with Supplementary Movie 3). Diffusive motion is characterized by its diffusion coefficient (*D*), which is proportional to the gradient of the plot in Fig. 1e. The length and width of the embryonic trunk increase linearly between E10.5 and E15.5 (Fig. 2a and Supplementary Table 2), this is accompanied by ∼6-fold increase in the number of melanoblasts between E10.5 and E11.5 (as identified by *Dct::lacZ* staining, Fig. 2b,c) and an increase in the mean melanoblast density in the mid-trunk region from ∼200 to ∼700 cells per mm^2^ between E12.5 and E15.5 (Fig. 2d). We examined the relationship between cell density and *D* and found a strong negative correlation (Fig. 2e); melanoblasts diffuse faster when they are less densely packed. We also examined melanoblast proliferation by measuring the frequency and length of mitotic events (Supplementary Movie 5). The mean melanoblast M-phase length (*T*_m_) was independent of cell density, but the proportion of mitotic cells (*P*_mc_) was strongly negatively correlated with density and the cell cycle time (*T*_c_*=T*_m_/*P*_mc_)^37^,^38^,^39^ was consequently strongly positively correlated with increasing density (Fig. 2f and Supplementary Fig. 1d,e).

### Stochastic modelling of melanoblast colonization

We hypothesized that undirected melanoblast movement and proliferation, in tandem with tissue growth (Fig. 2a and Supplementary Table 2) are sufficient for melanoblast colonization and that this simple mechanism can explain the patterns observed in chimeras, individually labelled clones and Kit mutants. We used our observations to parameterize a stochastic model of melanoblast colonization of the trunk (Methods). Our modelling framework only considers the growth of the trunk region and its colonization by the migrating melanoblast population. The domain is limited axially to the region between and not including the limb buds and encompasses the complete dorsoventral length (Fig. 2a and Methods). We assume that no new melanoblasts are specified after E10.5 and therefore that the growing melanoblast population is produced solely by the proliferation of this founder population. Melanoblasts migrate first within the developing dermis between E10.5 and E12.5, and subsequently within the epidermis and dermis between E12.5 and E15.5. We collectively refer to the dermal and epidermal layers that can support melanoblast survival as the dorsoventral integument (DVI; Methods, Supplementary Fig. 2) and assume that melanoblast behaviour in these compartments is equivalent. We describe above through analysis of cell orientations in *Dct::lacZ* embryos and time-lapse experiments that there is no directed migration in either compartment. In our simulations we employed an agent-based, discrete-space random-walk model on a growing two-dimensional lattice employing volume exclusion, whereby at most one agent (melanoblast) occupies each square lattice site, and melanoblasts cannot migrate or proliferate into occupied sites^40^. The stochastic events are simulated using the Gillespie algorithm (Methods)^41^.

**Figure 3:**
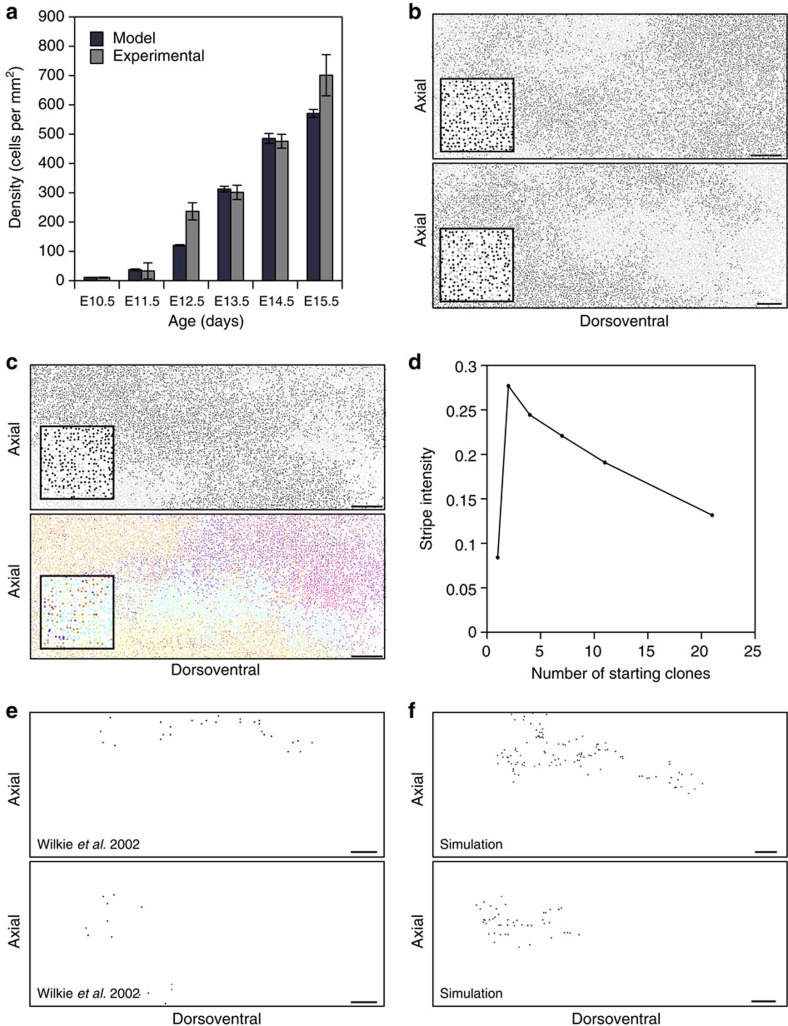
Random migration and proliferation can generate chimeric and diffuse rare clones. (**a**) Comparison of the mid-domain melanoblast densities at E10.5 (*n*=25), E11.5 (*n*=11), E12.5 (*n*=8), E13.5 (*n*=8), E14.5 (*n*=32) and E15.5 (*n*=7) with results from the stochastic model (model data are averaged over 100 repeats). (**b**) Two examples of the final time point of discrete simulations (*t*=5 days, equivalent to E15.5) using random labelling of the initial cells in two colours (black or light grey, inset) to form balanced chimeric patterns (analogous to mouse aggregation chimeras). (**c**) Comparative plots (*t*=5 days) of the same simulation using two clonal subtypes or 21 clonal subtypes. The coherent patches seen in the two-colour plots are composed of multiple coalescent subclones. (**d**) When only two clonal subtypes are present stripes are most apparent. As the number of subtypes is increased ‘stripe intensity' is reduced (where stripe intensity is defined as the dominant frequency emerging from the mean DFFT of the intensity profile of 100 simulations). (**e**) Two examples of rare *Dct::lacZ* revertant melanoblast clones generated using the *Dct::laacZ* mouse model described in Wilkie *et al*.^22^ (replotted here for consistency—clones shown in black). Redrawn with permission from Development^22^. (**f**) Patterns qualitatively similar to those in **e** generated by randomly labelling a single cell during a simulation and tracing all its progeny (*t*=5 days, clones shown in black). In **b**,**c**,**e** and **f** each plot represents one side of the embryo extending from the dorsal most aspect on the left to the ventrum on the right. Scale bars, 500 μm in all cases. Error bars in **a**=s.e.m.

Using the parameters generated from our experimental observations (Methods and Supplementary Table 3) our model was able to replicate the relationships between cell density, the diffusion coefficient and cell cycle time described above (red lines in Fig. 2e,f). It predicts colonization of the growing domain. That is, the averaged cell density in the model at all stages of embryonic development closely fits our experimental data (Fig. 3a). Examples of domain colonization are provided in Supplementary Movie 6 and Supplementary Fig. 4a.

### Chimeric and mosaic patterns formed by a common mechanism

**Figure 4:**
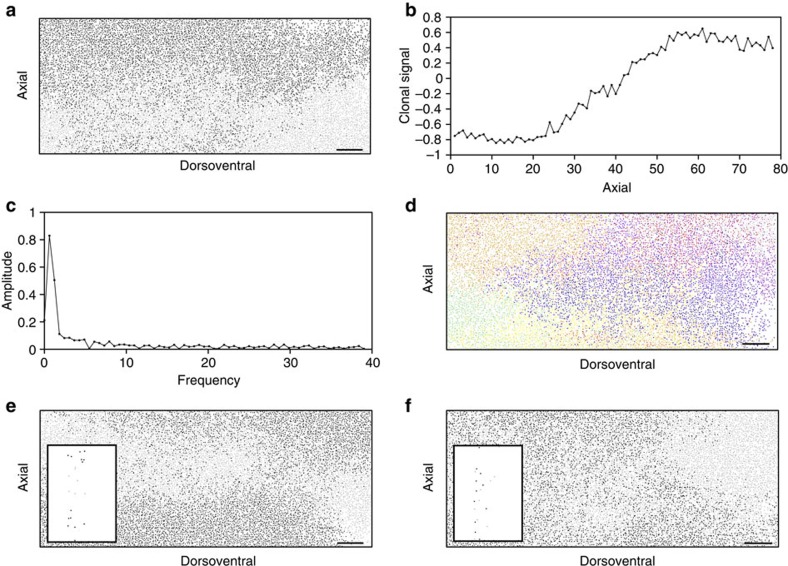
Segregation of clonal subtypes in the initial pattern determines stripe intensity. (**a**) A stripe-like pattern from a single simulation of the discrete model initialized with agents of two clonal subtypes (black and light grey) in equal proportions (analogous to a balanced mouse aggregation chimera). (**b**) The axial profile of the agent intensity for the simulation depicted in **a** reflecting the axial change in the dominant subtype (Methods). (**c**) Amplitude spectrum from a DFFT of the clonal signal (shown in **b**). (**d**) Data from the simulation in **a** plotted as individual clones. Increasing the number of clonal subtypes in the initial conditions from 2 to 21 removes the appearance of the stripe-like pattern and reveals the extent of mixing of the individual subclones. (**e**) A simulation in which the initial pattern containing two clonal subtypes has been deliberately well segregated in the initial conditions (black and light grey cells, shown in inset). Segregation of the initial subpopulations promotes dorsoventral stripe formation. (**f**) A simulation in which the initial pattern containing two clonal subtypes has been deliberately evenly mixed in the initial conditions (black and light grey cells, shown in inset). Even mixing of the initial subpopulations inhibits dorsoventral stripe formation, however areas that contain a single dominant clonal subtype are still present. Each plot in **a**,**d**,**e** and **f** represents one side of the embryo extending from the dorsal most aspect on the left to the ventrum on the right. Scale bars, 500 μm in all cases.

**Figure 5:**
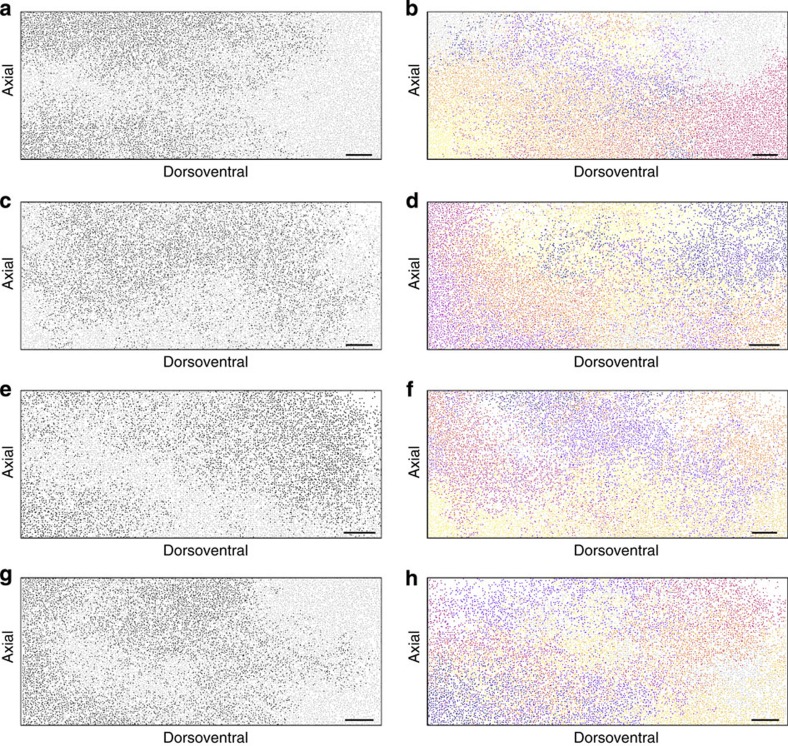
Extensive mixing of individual clones in the discrete model. (**a**–**h**) Example patterns from single simulations of the discrete model initialized with either agents of 2 clonal subtypes in equal proportions ((**a**–**g**) analogous to a balanced mouse aggregation chimera) or with 21 differently coloured subtypes (**b**–**h**). The left-hand and right-hand plots are generated from a single simulation. Each plot represents one side of the embryo extending from the dorsal most aspect on the left to the ventrum on the right. Scale bars, 500 μm in all cases.

To explore the relationship between mixing, tissue growth and proliferation we simulated rare melanoblast clones (by mimicking *in silico* the experiments of Wilkie *et al*.^22^; Methods, Fig. 3e–f), and chimeric patterns (two coloured and multi coloured; Fig. 3b–c and Methods). Our analysis of simulation data suggests that the prominence of stripe-like patterns (an emergent property of the model—Figs 3b,c and 5; column a–g) is influenced by the number of clonal subtypes at the start of a simulation and the degree to which they are mixed. We find that stripes are most apparent when we initialize with only two clonal subtypes where a single, low frequency corresponding to the two-striped pattern is clearly identifiable in the discrete fast Fourier transform (DFFT) of the clonal signal (Fig. 4c and Methods). This corresponds to most experimental patterns in mice consisting of only two colours (Fig. 3d, Methods and Fig. 4). Our analysis suggests these stripes are composed of multiple subclones of like genotype, formed by the stochastic coalescence of like clones and that, contrary to received wisdom, stripe formation does not proceed through the directed migration of coherent or descendent clones^42^ but rather stripes form from a favourable arrangement of like clones in the initial conditions that are elongated dorsoventrally by the bias in growth in that direction (Supplementary Table 2). Increasing the number of differently labelled clonal populations removes the stripe-like pattern in the chimeras (Figs 4d and 5). For example, compare the panels in Figs 3c and 5a–h, showing two-colour and multicolour depictions of single simulations. The dominant patches of colour in the first panel (Fig. 3c, top; and Fig. 5a–g) are composed of multiple subclones in the second (Fig. 3c, bottom; and Fig. 5b–h). Clones originating from what was previously the same labelled subpopulation (but not progeny of the same initial cell) now appear distinct from each other. If subpopulations are not well segregated initially then the formation of dorsoventral stripes is also less likely. Examples of well-segregated and poorly segregated initial subpopulations and the resulting patterns are shown in Fig. 4e,f, respectively. When rare melanoblast clones (Fig. 3e,f) are simulated (Methods) and plotted individually, they appear qualitatively similar to the patterns previously described in *Dct::laacZ* embryos^22^, and are therefore reconciled with stripe formation in our model.

### Stochastic evolution of dominant lineages in the model

Using our discrete model we observed a weak selection bias towards a small number of dominant lineages. Typically the two most dominant lineages (of the 21 initially specified) in a given simulation accounted for around 25% of the total number of melanoblasts at the end of the simulation (Supplementary Fig. 3b). To explore this effect further we initialized our discrete model with the movement and proliferation parameters employed in this study but with the same initial agent distribution and domain size described by Cheeseman *et al*.^26^,^27^—a two-dimensional square lattice of size *L*_*x*_(*t*)=50 by *L*_*y*_(*t*)=50 in which the 10 left-most columns are fully occupied. Using these conditions we observed a strong selection bias towards a small proportion of clonal subtypes. On average, two agents from the original 500 contributed over 25% of the final agent density (Supplementary Fig. 3c,d). This suggests that selection bias is heavily influenced by the initial conditions. The relatively sparsely packed distribution of melanoblasts at E10.5 would likely result in a weaker selection bias than in the developing intestine where pre-enteric neural crest cells are more numerous and tightly packed in the foregut before they embark on their colonization^43^.

### Belly spots arise from reduced proliferation in Kit mutants

**Figure 6:**
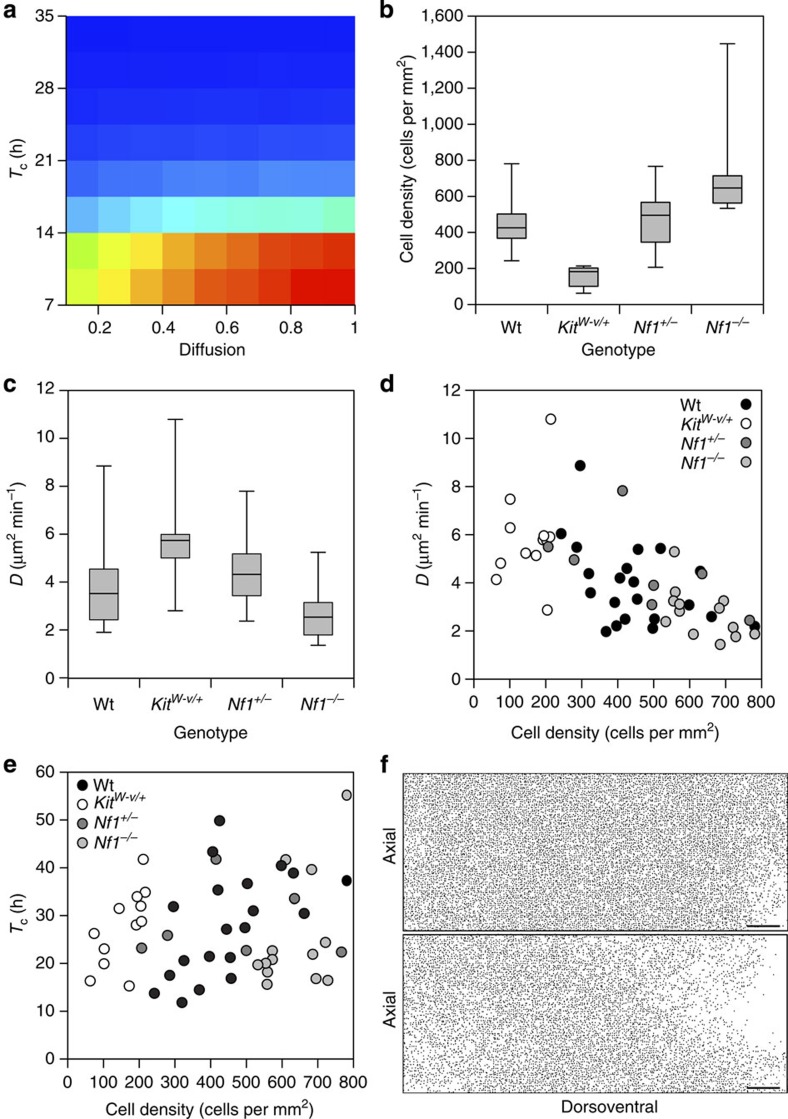
Reduced proliferation results in belly-spot formation. (**a**) A heat map generated from a parameter sweep comparing colonization in the model for different values of diffusion (normalized with respect to *D*_0_, the diffusion coefficient used in our simulations) and cell cycle time (*T*_c_). The model is substantially more sensitive to changes in *T*_c_ than in diffusion as indicated by the red region (blue: low probability of colonization; red: high probability of colonization). In all, 100 repeats of the model were performed for each combination. (**b**) Mid-trunk melanoblast densities of *Kit*^*+/+*^; *Nf1*^*+/+*^ (*n*=20), *Kit*^*W-v/+*^; *Nf1*^*+/+*^ (*n*=12), *Kit*^*+/+*^; *Nf1*^*+/−*^ (*n*=7) and *Kit*^*+/+*^; *Nf1*^*−/−*^ (*n*=14) embryos. Melanoblast density is reduced in *Kit*^*W-v/+*^; *Nf1*^*+/+*^ mice and is increased in *Kit*^*+/+*^; *Nf1*^*−/−*^ mice. One-way analysis of variance (ANOVA) *P*<0.0001, Tukey's honest significant difference test (Tukey's HSD) *P*<0.001 in both cases. (**c**) Melanoblast diffusion coefficients (*D*) for *Kit*^*+/+*^; *Nf1*^*+/+*^ (*n*=21), *Kit*^*W-v/+*^; *Nf1*^*+/+*^ (*n*=12), *Kit*^*+/+*^; *Nf1*^*+/−*^ (*n*=7) and *Kit*^*+/+*^; *Nf1*^*−/−*^ (*n*=14) embryos. Diffusion is increased in *Kit*^*W-v/+*^; *Nf1*^*+/+*^ mice despite the failure of the melanoblast population to completely colonize the dorsoventral domain (one-way ANOVA *P*<0.0001, Tukey's HSD *P*<0.01). (**d**) Plot of *D* against density for *Kit*^*+/+*^; *Nf1*^*+/+*^ (*n*=20), *Kit*^*W-v/+*^; *Nf1*^*+/+*^ (*n*=12), *Kit*^*+/+*^; *Nf1*^*+/−*^ (*n*=7) and *Kit*^*+/+*^; *Nf1*^*−/−*^ (*n*=14) embryos. Pearson's product-moment correlation indicates a significant negative association (*r*=−0.62, df=51, *P*<0.0001). (**e**) Plot of *T*_c_ against cell density for *Kit*^*+/+*^; *Nf1*^*+/+*^ (*n*=19), *Kit*^*W-v/+*^; *Nf1*^*+/+*^ (*n*=12), *Kit*^*+/+*^; *Nf1*^*+/−*^ (*n*=7) and *Kit*^*+/+*^; *Nf1*^*−/−*^ (*n*=14) embryos. Pearson's product-moment correlation indicates no association (*r*=0.26, df=50, *P*=0.058). (**f**) An increase in *T*_c_ (from 7 to 10 h) results in a ventral belly spot in our simulations qualitatively similar to the pattern observed in *Kit*^*W-v/+*^ mice (*t*=5 days, equivalent to E15.5). Each plot represents one side of the embryo extending from the dorsal most aspect on the left to the ventrum on the right. Scale bars in **f**, 500 μm. Wt, wild type. Whiskers in b, c=maximum and minimum of all data. The boxes enclose the 2nd and 3rd quartiles.

Belly-spot formation has long been proposed a product of either altered proliferation or migration or both. To compare these effects in the model we performed a parameter sweep comparing the extent of colonization at different values of *D* and *T*_c_. This revealed that our stochastic model is markedly more sensitive to changes in proliferation rate than in the diffusion coefficient (Fig. 6a). Nf1 is a GTPase-activating protein that negatively regulates Ras activity downstream of Kit signalling. Ablation of *Nf1* results in constitutive Ras activity and increased proliferation^44^. To investigate the role of *Kit* signalling in melanoblast colonization we performed live-imaging experiments at E14.5, labelling melanoblasts on a *Kit*^*W-v/+*^ background^45^ or ablating *Nf1* (Methods) to generate melanoblast-specific *Nf1*^*−/−*^ or *Nf1*^*+/−*^ genotypes. We found a lower density of melanoblasts in the E14.5 trunk of *Kit*^*W-v/+*^ mice whilst the density was increased in *Nf1*^*−/−*^ mice implying a reduction and an increase in melanoblast proliferation, respectively (Fig. 6b). However contrary to expectations we observed a significantly higher rate of diffusion in *Kit*^*W-v/+*^ mutants. Diffusion was reduced in *Nf1*^*−/−*^ animals, but the change was not significant (Fig. 6c). A plot of density against *D* (Fig. 6d) indicates that for their given densities *Kit*^*W-v/+*^ and *Nf1*^*−/−*^ melanoblasts behave in a similar manner to wild type (the negative association with density observed in wild type is preserved when the data are combined; Fig. 6d) suggesting that the change in diffusion rates are a consequence of changes in cell density.

**Figure 7:**
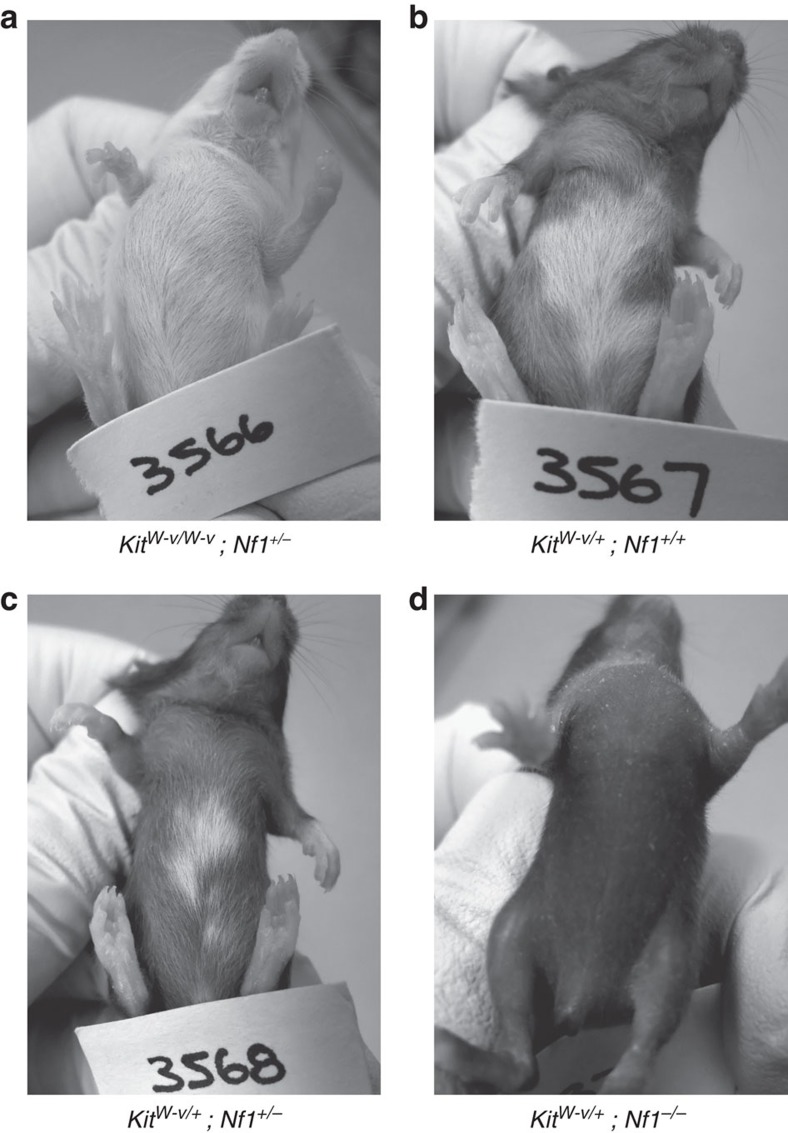
Deletion of *Nf1* rescues the belly spot in *Kit* mutants. (**a**) No pigmentation is present in homozygous *Kit*^*W-v/W-v*^ mutants on an *Nf1*^*+/+*^ or *Nf1*^*+/−*^ background (pictured) due to a complete lack of melanocytes in the adult. (**b**) Heterozygous *Kit*^*W-v/+*^ mice on an *Nf1*^*+/+*^ background exhibit a large ventral belly spot due to a failure of complete melanoblast colonization of the developing epidermis. (**c**) The belly spot is partially rescued in heterozygous *Kit*^*W-v/+*^ mice on an *Nf1*^*+/−*^ background. (**d**) The belly spot is completely rescued in heterozygous *Kit*^*W-v/+*^ mice on an *Nf1*^*−/−*^ background.

Plotting *T*_c_ against density for *Kit*^*W-v/+*^, *Nf1*^*+/−*^ and *Nf1*^*−/−*^ mutants and wild type we find that for a given density *Kit*^*W-v/+*^melanoblasts proliferate more slowly than would be expected and *Nf1*^*−/−*^ more quickly (negating the positive wild-type association with density when the data are combined; Fig. 6e). We calculated a corrected cell cycle time (as *T*_c_/density multiplied by 200 cells per mm^2^) this revealed a longer cell cycle time in *Kit*^*W-v/+*^ melanoblasts (Supplementary Fig. 1g), and is the likely causal factor in belly-spot formation. We used our model to test this conclusion with the assumption that as melanoblasts are specified from the neural crest in a Kit-/Kitl-independent manner^1^ there would be equal numbers in wild-type and *Kit*^*W-v/+*^ melanoblasts in the MSA at E10.5. Accordingly, increasing cell cycle time in the model results in the failure of melanoblasts to fully populate the domain and produces a white belly spot that mimics that seen *in vivo* (Fig. 6f, Supplementary Fig. 4 and Supplementary Movie 7). As the cell cycle time is increased, belly spots are increasingly observed, followed, at longer times, by dorsal spots along with larger belly spots. This is consistent with existing mutant phenotypes, small spots are usually confined to the belly, whilst larger belly spots are often accompanied by dorsal spotting, such as in mutants of *Rac1* (ref. 46) or *Magoh*^47^. Furthermore, despite there being no change in cell motility on the *Nf1*^*−/−*^ background, loss of Nf1 rescues the belly spot in *Kit*^*W-v/+*^ individuals (Fig. 7a–d).

## Discussion

In summary we show experimentally that migrating melanoblasts do not have preferred directionality, but rather diffuse and proliferate throughout the developing skin in a density-dependent manner. Furthermore, repulsive events between adjacent melanoblasts do not seem to contribute to their dispersal, and the well-characterized ‘follow my leader' behaviour observed in cranial neural crest populations^28^ was not observed. The spatial distribution of melanoblasts appears random above a distance of ∼28 μm. Consistent with this, occasional polygonal regions of exclusion are observed (Supplementary Movie 4), reflecting the underlying packing of the epidermal keratinocytes. We demonstrate experimentally that melanoblasts carrying the *Kit*^*W-v*^ mutation do not migrate more slowly, but instead diffuse in a density-dependent manner similar to wild-type melanoblasts. The mutant cells, however, proliferate more slowly than expected, and our modelling suggests that this is the likely cause of the white belly spot. Many mutations result in white belly spots^48^, often in genes that would not be expected to affect cell motility such as the translation initiation factor *Eif3c* (ref. 49), the ribosomal protein *S7* (ref. 50) and the chromatin-modifying enzyme *Mysm1* (ref. 51). We suggest that in many cases the white spotting is due to defects in melanoblast proliferation rather than motility.

Using stochastic individual-level modelling we have examined the importance of density-dependent diffusion and proliferation for colonization of the DVI and conclude that colonization is most sensitive to changes in proliferation. This is in agreement with Zhang *et al*.^29^ who explored the interaction between neural crest migration and proliferation using an on-lattice model for the colonization of the gut by enteric ganglia progenitors^29^. One weakness of our model is that it assumes that melanoblast behaviour is equivalent in the relatively sparsely packed three-dimensional dermal environment between E10.5 and E12.5, and in the more tightly packed two-dimensional epidermal environment between E12.5 and E15.5. Experimentally, we demonstrate that this is qualitatively the case but there will certainly be minor differences. The on-lattice approach we use is more appropriate for the latter of these scenarios. However, to represent these two environments separately would require a computationally intensive hybrid model and a number of new and potentially inaccessible parameters, which would complicate the model and hamper the investigation of the patterning questions we chose to address. Our model assumes that all melanoblasts arise by proliferation of the differentiated melanoblasts present at E10.5. This may not be the case as further cells fated to be melanoblasts may differentiate after E10.5. Another source of melanoblasts may be from Schwann cell precursors (SCPs) emanating from the dorsal ramus from E12.5 onwards as has been proposed by Adameyko *et al*.^52^. However, as the lineage tracing approach that identified these cells has been questioned^53^,^54^ and we have no access to the key parameters of their possible behaviour, incorporating Schwann cell precursor-derived melanoblasts into the present model is not feasible.

Cheeseman *et al*.^26^,^27^ investigated the dominance of sub-lineages in a lattice-based discrete model. They found that in many cases the progeny of two cells (of the 500 they initialized) could contribute in the order of 25% of the cells in the final population. This effect was mediated by a process of sequential isolation of individual lineages deprived of space to proliferate into^26^,^27^. This stochastic drift in clone size has been demonstrated experimentally and explored mathematically in the mouse intestine^55^,^56^,^57^. Selection of dominant lineages is relatively weak in our simulations of melanoblast domain colonization owing to the more diffuse initial conditions. More cells are able to establish a significant lineage because they have the required space to proliferate initially and consequently fewer lineages become spatially isolated. This implies that the stripes seen in our model are predominantly formed by the coalescence of multiple like-coloured subclones, and not by the presence of dominant lineages. Furthermore, in our model, the domain grows in both the dorsoventral and axial directions, whereas in Cheeseman *et al*.^26^,^27^ domain growth is only in the dorsoventral direction or is absent. The two-dimensional growth in our model further reduces the role of dominant lineages since cells, which may previously have been isolated, can gain space into which they may proliferate through domain growth events. Our modelling shows that the generation of rare clones^22^ and chimeric patterns^19^ can proceed through a common mechanism employing tissue expansion and density-dependent movement and proliferation. Further experimental clonal analyses, using stochastic labelling methods such as brainbow/confetti^57^,^58^, are required to explore whether our predictions of the behaviour of melanoblast subclones are accurate.

In conclusion, belly-spot formation, chimeric patterns and diffuse clonal patterns are all explained by a simple model incorporating random melanoblast migration with proliferation, in conjunction with domain growth, during the course of colonization. Importantly, colonization and the observed phenotypes are produced without the need for more complex cell–cell interactions or extracellular signals and this has broader implications for cell behaviour in other NCSC lineages and their associated neurocristopathies.

## Methods

### Animal models

All animal work was approved by a University of Edinburgh Internal Ethics Committee and was performed in accordance with the institutional guidelines under licence by the UK Home Office (PPL 60/4424 and PPL 60/3785). Mice were maintained in the animal facilities of the University of Edinburgh. Mouse lines containing the transgenes or modified alleles *Dct::lacZ* (generated in-house)^6^, *R26R-YFP* (kindly provided by Prof L. Smith, The University of Edinburgh)^59^, *Tyr::CreA* and *Tyr::CreB* (kindly provided by Prof. L. Larue, Institute Curie, Paris)^60^, *Nf1*^*flox*^ (obtained from the National Cancer Institute, Mouse Repository, Frederick, USA)^61^ and *Kit*^*W*-v^ (obtained from the Medical Research Council, Harwell, UK)^45^ were genotyped according to published methods. *Pmel*^*CreERT2*^ mice (unpublished, generated in-house) were genotyped using the PCR primers Pmel_For (5′- GGGTAAAGAAGAGGGGAGAGG -3′), Pmel_Rev (5′- GGGATGTTCCATCACCTTCA -3′) and CreERT2_Rev (5′- AGGCAAATTTTGGTGTACGG -3′) to distinguish between targeted and wild-type alleles. Animals used to investigate adult belly spots were male progeny from a cross between *Nf1*^*+/flox*^; *Kit*^*W-v/+*^ males and a *Tyr::CreA*^*Tg/Tg*^; *Nf1*^*+/flox*^; *Kit*^*W-v/+*^ females on a mixed genetic background. Only male animals were considered as the *Tyr::CreA* transgene is X-linked. *Tyr::CreA*^+ve^; *Nf1*^*flox/flox*^ animals were smaller than their litter mates. For live imaging of embryonic skin on a *Kit*^*W-v/+*^ background E14.5 progeny from a cross between *Tyr::CreB*^*Tg/Tg*^; *Kit*^*W-v/+*^ and *R26YFPR*^*Tg/Tg*^; *Kit*^*W-v/+*^ individuals or between *Tyr::CreB*^*Tg/Tg*^; *Kit*^*+/+*^ and *R26YFPR*^*Tg/Tg*^; *Kit*^*W-v/+*^ individuals were used on a mixed genetic background. No melanoblasts were observed in the back skin of E14.5 *Tyr::CreB*^*+ve*^; *R26YFPR*^*Tg+ve*^; *Kit*^*W-v/W-v*^ individuals. For live imaging of embryonic skin on a *Nf1*^*flox/+*^ and *Nf1*^*flox/flox*^ background E14.5 progeny from a cross between *Tyr::CreB*^*Tg/Tg*^; *Nf1*^*flox/+*^ and *R26YFPR*^*Tg/Tg*^; *Nf1*^*flox/+*^ individuals or *Tyr::CreB*^*Tg/Tg*^; *Nf1*^*flox/+*^ and *R26YFPR*^*Tg/Tg*^; *Nf1*^*flox/flox*^ individuals were used on a mixed genetic background. E14.5 *Tyr::CreB*^*+ve*^; *Nf1*^*flox/flox*^ individuals were viable and morphologically indistinguishable from their litter mates. To image melanoblast behaviour in whole embryos at E11.5 we examined the progeny of a cross between *Pmel*^*CreERT2 /CreERT2*^; *R26R-YFP*^*Tg/Tg*^ individuals on a mixed background. The pregnant mothers were given 8 mg of 4-hydroxytamoxifen per 40 g body weight gavage or injectionat E10.5. To investigate melanoblast numbers in fixed tissues *Dct::lacZ*^*Tg/Tg*^ and *Dct::lacZ*^*Tg/+*^ embryos were used resulting from crosses between combinations of *Dct::lacZ*^*Tg/+*^, *Dct::lacZ*^*Tg/Tg*^ and *Dct::lacZ*^*+/*+^ parents on CD1 background. Embryos used for optical projection tomography were F1 hybrids from a cross between the mouse strains C57Bl6 and CBA (obtained from Charles River Laboratories, UK).

### Embryonic skin culture and imaging of whole embryos

Embryonic skin culture was performed as described in Mort *et al*.^31^. Briefly, up to six cultures were imaged in parallel per experiment. Skin was sampled from the flank of E13.5, 14.5 and 15.5 mouse embryos. The dorsoventral position varied but was never taken at the ventral extreme. The skin samples were mounted on a clip filled with 1% w/v agarose (in PBS) and secured with suture thread. The clip was then inserted into a custom designed six-well chamber so that the skin was sandwiched against a lummox gas-permeable membrane (Greiner). The wells were filled with DMEM (no phenol red) supplemented with 1 × Glutamax (Gibco), 1% v/v penicillin/streptomycin and 10% v/v fetal calf serum. Whole E11.5 embryos were embedded in 1% w/v agarose (in PBS) in a large custom-made imaging clip so that the dorsal region of the flank was just protruding above the surface of the agarose. The clip was then inserted into a custom designed six-well chamber so that the protruding region of the embryo was pressed against a lummox gas-permeable membrane (Greiner). The wells were filled with DMEM (no phenol red) supplemented with 1 × Glutamax (Gibco), 1% v/v penicillin/streptomycin and 10% v/v fetal calf serum.

### X-Gal staining of embryos

X-Gal staining of *Dct::lacZ* embryos was performed as previously described^6^. Briefly, embryos were fixed in 4% w/v paraformaldehyde for varying times depending on developmental stage. They were then permeabilized in detergent wash solution (2 mM MgCl_2_, 0.05% w/v BSA, 0.1% w/v sodium deoxycholate and 0.02% v/v Igepal in 0.1 M sodium phosphate buffer; pH 7.3) before being stained overnight in X-Gal stain solution (5 mM K_3_Fe(CN)_6_, 5 mM K_4_Fe(CN)_6_, and 0.085% w/v NaCl with 0.1% w/v X-gal in detergent wash). Embryos were then subjected to further washes in detergent wash solution and then PBS before being post-fixed in 4% w/v paraformaldehyde (in PBS).

### Image acquisition

Time-lapse sequences of migrating melanoblasts in embryonic skin culture and whole embryos were captured on a Nikon A1R inverted confocal microscope using a × 20 objective, images were captured at 2-min (for skin) or 5-min (for embryos) intervals over the course of the experiment. A stage top environmental chamber was used providing 5% CO_2_ in air and maintaining a constant temperature of 37 °C. Images of X–Gal-stained *Dct::lacZ* embryos were captured on a Nikon macroscope using a ring light for illumination and a × 2 Nikon objective with × 2 optical zoom.

### Optical projection tomography

Optical projection tomography (OPT) was performed on Bouin's fixed mouse embryos at 1-day stages between E10.5 and E15.5. Samples were mounted in 1% low-melting-point agarose, dehydrated in methanol and then cleared overnight in BABB (1 part benzene alcohol:2 parts benzene benzoate). Samples were scanned using a Bioptonics OPT Scanner 3001 (Bioptonics, Edinburgh, UK) using a variety of fluorescent wavelengths to visualize tissue autofluorescence (excitation 425/60 nm/emission 480 nm and excitation 480/40 nm/emission 510 nm). Resultant scans were then reconstructed using proprietary software (nRecon/Skyscan, Belgium).

### Image analysis and cell tracking

All image analysis tasks were performed using custom written macros for the Fiji distribution of ImageJ^62^. All morphological and tracking procedures were carried out on segmented images using standard ImageJ routines. To automatically track melanoblasts in the time-lapse sequences a modified version of the wrMTrck plugin (http://www.phage.dk/plugins/wrmtrck.html) was used on segmented TIFF stacks, the script used relied on Gabriel Landini's morphology collection (http://www.mecourse.com/landinig/software/software.html). The tracking and morphology data generated by the procedure were recorded in a text file and used for the downstream analyses. The MSD of the melanoblast population was calculated from this data using the time ensemble averaging approach^63^, in a custom macro written for Fiji. Feret's angles from time-lapse sequences (E14.5) were calculated from the shape of the cell body after image segmentation. The angle of migration in X–Gal-stained samples (E11.5, 12.5, 13.5 and 15.5) was measured manually by drawing a line along the longest axis of each cell in ImageJ. To calculate cell densities for stages E10.5 and E11.5 (Fig. 3a) the total number of cells was divided by the area of the trunk calculated from our OPT data. For all other stages the mid-domain density was measured.

To analyse the cell cycle, only the first 4 h of each time-lapse was considered to minimize laser exposure. The mean M-phase length (*T*_m_) per time-lapse was calculated from the length of five mitotic events. The mean proportion of cells morphologically in M-phase (*P*_mc_) per time-lapse was calculated from five frames spaced 60 min apart over the first 4 h of each time-lapse. The cell cycle time (*T*_c_) for a given time-lapse was then calculated as *T*_c_*=T*_m_*/P*_mc_ (refs 37, 38, 39).

The PCF is a summary statistic that provides a quantitative measure of spatial patterning. The function is derived by normalizing the counts of the distances between pairs of agents^34^,^35^,^36^. It is therefore able to capture patterning and the length scale of individual objects. We applied the PCF with non-periodic pairwise distance counting to multiple microscopy images of melanoblasts in the developing epidermis at E14.5 using a custom Matlab script. To avoid issues associated with the image boundary (where cells had been lost due to image processing) we used only a 256 × 256 μm central portion of each image (we find similar results for alternative window sizes if the central portion is positioned sufficiently far away from the boundary).

### Measurement of domain expansion

To measure the expansion of the dorsoventral and axial domains of the trunk, OPT models were analysed using ImageJ/Fiji. Two measurements of the trunk circumference were made at the levels of the fore- and hindlimbs and averaged. For E10.5 where the umbilical hernia encompasses most of the axial width of the domain the region comprising the peritoneal membrane was excluded from the measurement as there is no dermal tissue at this level for melanoblasts to colonize. The dorsoventral length was defined as half the mean trunk circumference. Axial width was defined as the length between the hind- and forelimb junctions incorporating the curve of the domain at early stages (E10.5–E12.5).

### Statistics

All statistical tests were performed using the ‘R' statistics package, an open-source software package based on the ‘S' programming language (http://www.R-project.org). The Berman's^64^ test for a point process model was performed using the additional ‘spatstat' package (http://www.spatstat.org/). All correlations were explored by examining the Pearson's product-moment correlation coefficient. Comparisons between multiple groups were undertaken using a one-way analysis of variance. Subsequent pairwise comparisons were performed using a Tukey's honest significant difference test, which is corrected for multiple testing.

### Model framework

In our modelling framework we consider only the growth of the trunk region of the developing embryo between and not including the limb buds and its colonization by the migrating melanoblast population (Fig. 2a). We assume that melanoblast behaviour in the dermis (between E10.5 and E12.5) and the epidermis (between E12.5 and E15.5) is equivalent. The dermal and epidermal layers that can support melanoblast survival are collectively referred to as the DVI. We use an agent-based discrete random-walk model with volume exclusion on a two-dimensional square lattice of length *L*_*x*_(*t*) by *L*_*y*_(*t*) to model the DVI *L*_*x*_(*t*) represents the dorsoventral length of the domain at time *t* and *L*_*y*_(*t*) the axial length at time *t*. The lattice spacing is denoted Δ and time evolves continuously. Each agent (melanoblast) is assigned to a lattice site, from which it can move or place progeny into an adjacent site. Attempted agent movement or proliferation events occur with rate *P*_*m*_ or *P*_*p*_ per unit time, respectively. That is, *P*_*m*_*δt* is the probability of a given agent attempting to move in the next infinitesimally small time interval *δt* with events simulated as such using the Gillespie algorithm. If an agent attempts to move or proliferate into a site that is already occupied, the event is aborted.

### Modelling tissue expansion

To model domain growth we employ a stochastic ‘pushing' growth mechanism as described in Binder *et al*.^65^. The insertion of new lattice sites into the domain occurs with rates *P*_*ga*_ and *P*_*gd*_ per unit time, for growth in the axial and dorsoventral direction, respectively. When a ‘growth event' occurs in the dorsoventral direction (horizontal direction in Supplementary Fig. 2a), for each row of the lattice one new site is added in a column, which is selected uniformly at random. To accommodate the new sites, in each row, the sites to the right of the added site are shifted a distance Δ rightwards carrying their contents with them (that is, cells move with their sites). Likewise, for axial growth (in the vertical direction in Supplementary Fig. 2b) one new site is added to each column in a row, which is selected uniformly at random and the appropriate sites are shifted upwards. Growth is linear in both the dorsoventral and axial directions as evidenced by experimental data (Supplementary Table 2).

### Implementation of model

Movement, proliferation and growth events are modelled as exponentially distributed ‘reaction events' in a Markov chain. Specifically we use the ‘Gillespie' Monte Carlo simulation algorithm to simulate realizations of our model system. Each realization represents 5 days of real time from E10.5 to E15.5. We implement zero-flux boundary conditions on all boundaries in our discrete model. This represents the assumption that melanoblast efflux is balanced by melanoblast influx at the boundaries of the domain.

### Modelling parameters from experimental data

*Lattice spacing*. The lattice spacing is chosen as Δ=38 μm. This implies that a single agent excludes a volume of 1,444 μm^2^, which is a realistic estimate for the size of a melanoblast. A completely colonized model domain (that is, every site in the computational domain is occupied by an agent) has a density of ∼692 cells per mm^2^. Our experiments have established that the mean (±95% confidence interval (CI)) density of a ‘colonized' domain at E15.5 is 701.21±137.70 cells per mm^2^ (main text, Fig. 2d).

*Domain size and growth rates*. Linear isotropic domain growth for the axial and dorsoventral domains was defined from morphological analysis of optical projection tomographs at embryonic stages between E10.5 and E15.5 (Supplementary Table 2). We initialize the domain as a rectangle of length 1,178 μm in the dorsoventral direction and 1,634 μm in the axial direction (corresponding to 31 lattice sites by 43 lattice sites, respectively). Although the domain grows stochastically, we employ constant growth rates *P*_*ga*_=0.00526, min^−1^ and P_gd_=0.0246, min^−1^ in the axial and dorsoventral directions, respectively, such that the mean-field growth in each direction is linear and matches with the experimentally measured linear domain growth relationship.

*Initial number and position of cells*. We defined the number of progenitor melanoblasts by counting the melanoblasts in the trunk of *Dct::lacZ* embryos at E10.5; a mean (±95% CI) of 20.32±5.95 melanoblasts (Fig. 2c). In our *Dct::lacZ* embryos we noted an under-representation of melanoblasts in the centre of the trunk region, although not always clear at E10.5 this was most striking at E11.5 (Supplementary Fig. 1c). We therefore weighted our initial distribution in a similar manner, initializing 21 agents such that on average one-third are between sites 12 and 32 of the axial axis, and the remaining two-thirds are evenly distributed between sites 1 and 11, and 33 and 43 corresponding to a slight under-representation in the middle of the axial axis. These agents are distributed so that 95% are between sites 8 and 17 of the dorsoventral axis. All agents are distributed between sites 8 and 19 of the dorsoventral axis.

*Diffusion rate*. As described in the main text we determined experimentally a density-dependent relationship between melanoblast diffusion and local density (main text, Fig. 2e). To determine the same relationship in our model we track agents moving on a 646 × 646-μm domain (corresponding to 17 × 17 sites) with periodic boundary conditions. This domain size corresponds, approximately, to the field of view of the microscope used to collect the experimental data on melanoblast movement. At *t*=0 in the simulation, a number of agents (from 1 to 289, representing all possible non-zero agent densities) are initialized with positions chosen uniformly at random throughout the domain. These agents are allowed to move (but not to proliferate, so as to keep the density constant) as described above for a simulation duration equivalent to 400 min of real time. This process is repeated 100 times for each agent density to guarantee enough data for an accurate representation of the MSD of the population. In each simulation the resulting agent tracks (excluding, for those agents that crossed a boundary, the portion of their tracks after their first boundary crossing event, since the tracks of these agents would be lost in our experimental system) are used to characterize the MSD as described in the main text. To determine the movement rate *P*_m_, we compare the relationship between density and effective diffusion coefficient for the experimental data to those for the model for a range of different values of *P*_m_. We chose the value of *P*_*m*_ that gives the best fit (smallest least squares error, *l*^2^ norm). This value *P*_*m*_ is given in Supplementary Table 3.

*Proliferation rate*. We defined the maximum proliferation rate by counting the number of melanoblasts in the trunk of *Dct::lacZ* E10.5 and E11.5 embryos. We found a mean (±95% CI) of 20.32±5.95 melanoblasts increasing to 151.09±27.95 melanoblasts in the first 24 h (Fig. 2c). To estimate a maximum doubling time for this period we used the mean cell number at E10.5 (–95% CI=14 cells) and the mean cell number at E11.5 (+95% CI=179 cells) implying a mean dermal doubling time of 6.6 h. We therefore chose a maximum possible population doubling time in the model of 7 h.

*Simulation of rare clonal patterns*. To investigate rare clones, at a time point chosen uniformly at random during the simulation, we chose one of the agents, from amongst all the agents that populate the domain at that time, with equal probability. This agent is marked and all the agent's progeny inherit the same mark. At the end of the simulation all marked agents are plotted in a different colour to the non-marked agents resulting in a diffuse rare clonal pattern as seen in Supplementary Fig. 3a and in Fig. 3f.

*Identification of stripe-like patterns in the model*. To investigate chimeric stripe-like patterns in our discrete model we initialized our simulations with two distinctly labelled agent subpopulations and tracked the positions of their progeny over time (Fig. 4a). When the simulation was complete, we assigned the value +1 (associated with light grey cells) to one of the agent types and −1 (associated with black cells) to the other (while empty lattice sites are assigned the value 0). We then averaged the values associated with the lattice sites on each row. This provides a measure of the proportion of each agent colour in each row, which we call the ‘clonal signal' (Fig. 4b). We repeat this process for each of the possible divisions of the 21 initial cells into two non-overlapping subsets, which we call a ‘clonal ratio'. For instance to investigate the patterning of a single clone we label one clone with +1s and remaining 20 clones with −1s. In this way we can investigate the pattern formed by a single clone amongst 21 differently labelled clones. Similarly, to investigate the pattern formed by ∼11 distinctly labelled clones we label two of the randomly selected clones with 1s and 19 with −1s. To investigate the effect of having only two different clonal labels, we label approximately half (10 or 11) of the randomly selected clones with 1s and the other half with −1s.

To identify the presence of stripe-like patterns in our simulations we apply the DFFT to our intensity profiles. We repeat this process 100 times and generate an average DFFT for each different initial clonal ratio (Fig. 4c). For simulations without stripe-like patterns (that is, when the agents from different subpopulations are well mixed) no dominant frequency is clearly identifiable. However, in the case where different agent subpopulations are not well mixed and have formed dorsoventral stripes, a single, low frequency is identifiable that relates directly to the periodicity of the stripes in the simulation. A dominant frequency (corresponding to the maximum value of the averaged DFFT) can be identified for each initial clonal ratio. We call this dominant frequency the ‘stripe intensity' (Fig. 3d). This method allows us to systematically identify the presence of stripes.

## Additional information

**How to cite this article:** Mort, R. L. *et al*. Reconciling diverse mammalian pigmentation patterns with a fundamental mathematical model. *Nat. Commun.* 7:10288 doi: 10.1038/ncomms10288 (2016).

## Supplementary Material

Supplementary Information Supplementary Figure 1-4, Supplementary Tables 1-3 and Supplementary Reference

Supplementary Movie 1 Supplementary Movies 1 and 2: E11.5 melanoblast behaviour. Whole embryo culture showing melanoblast dermal behaviour on the dorsolateral pathway. Melanoblasts migrate constantly and apparently randomly and are seen to move both dorsoventrally and ventrodorsally. The arrow indicates the dorsoventral axis. D = dorsal, V = ventral.

Supplementary Movie 2 (same as Supplementary Movie 1)

Supplementary Movie 3 E14.5 melanoblast behaviour. Embryonic skin culture showing melanoblast behaviour in the epidermis. Melanoblasts migrate constantly and apparently randomly pausing only to undergo mitosis before continuing their otherwise constant movement.

Supplementary Movie 4 Melanoblasts migrate along their Feret's diameter. A field of migrating melanoblast at E14.5. The Feret's diameter of the cell body is drawn in cyan. Melanoblasts can be see to migrate along this axis. An example track is highlighted in Red. The Feret's diameter is therefore a reasonable proxy for the direction of migration.

Supplementary Movie 5 Measurement of melanoblast mitosis length. An individual melanoblast transiting M-Phase. M-phase was defined morphologically as the period from which the cell is round until the end of cytokinesis as indicated by the cyan outline.

Supplementary Movie 6 Colonisation with the discrete model. A representative simulation of melanoblast colonisation generated using the discrete model with wildtype parameters (7-hour cell cycle time). The simulation lasts for 5 days starting at E10.5 and finishing at E15.5. Left and right domains are shown with the ventrum in the centre of the image.

Supplementary Movie 7 Non-colonisation with the discrete model. A representative simulation of melanoblast colonisation generated using the discrete model with reduced proliferation (10-hour cell cycle time). The simulation lasts for 5 days starting at E10.5 and finishing at E15.5. Reduced cell densities can be observed at the end of the simulation with a clear unpopulated patch at the most ventral position. Left and right domains are shown with the ventrum in the centre of the image.
