# Supplementary material for: Reconciling diverse mammalian pigmentation patterns with a fundamental mathematical model
Source: Nat Commun. 2016 Jan 6;7:10288. doi: 10.1038/ncomms10288 (PMC4729835; doi:10.1038/ncomms10288)
Supplement: Supplementary Information — Supplementary Figure 1-4, Supplementary Tables 1-3 and Supplementary Reference [file ncomms10288-s1.pdf]

Supplementary Figures

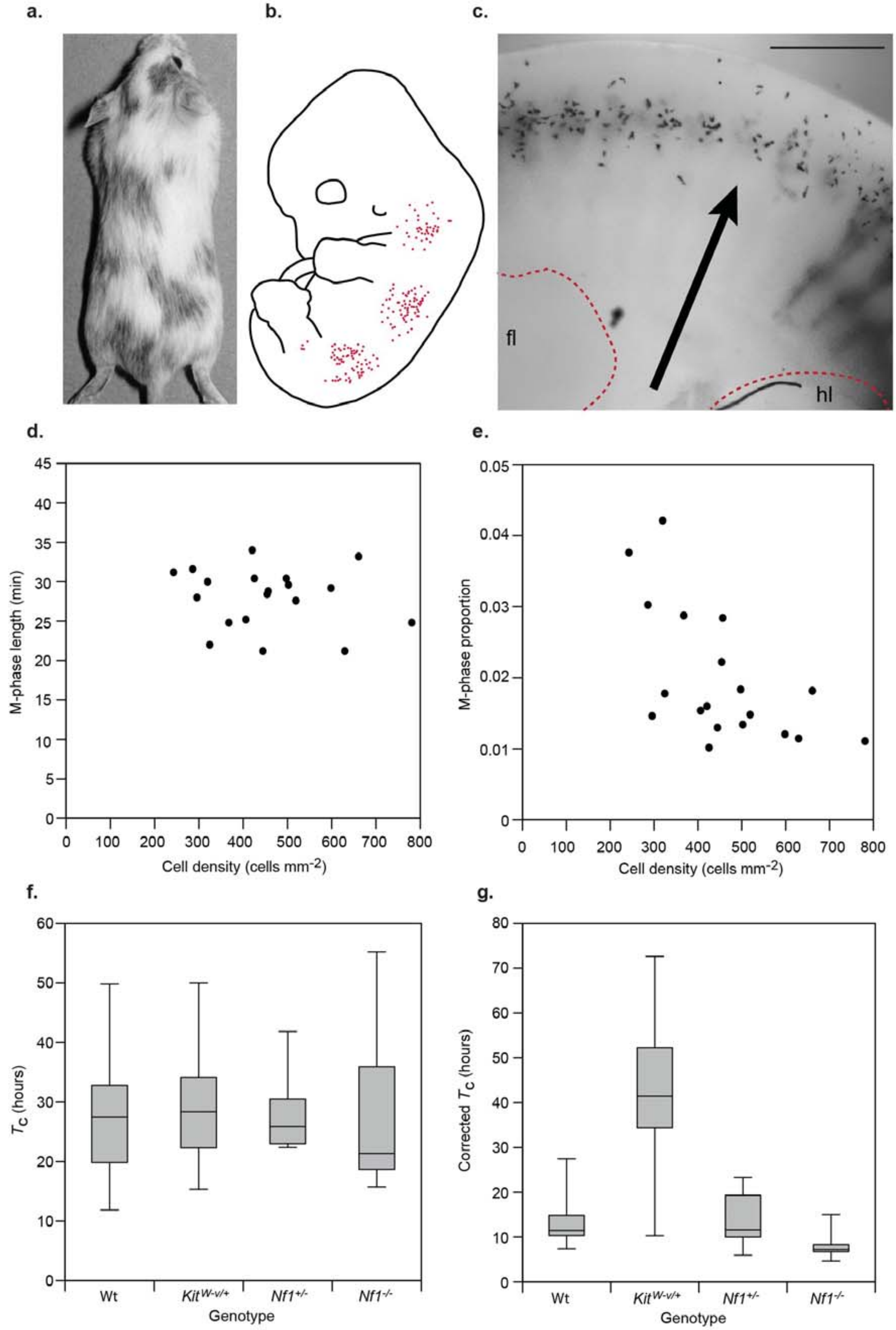

Supplementary Figure 1. Melanoblast patterning and cell cycle. a: A chimaera made by aggregation of cleavage stage embryos of two genotypes: pigment producing and non pigment producing. The patches of pigmentation extend dorsoventrally and generally do not cross the dorsal midline. This can be taken as evidence of the dorsoventral migration in the developing embryo. **b:** Camera lucida generated image of rare (*Dct::laacZ)* melanoblast clones in the developing embryo at E13.5 exhibiting high levels of axial mixing (Adapted (re-drawn) with permission from Development, Wilkie *et al.*[1]. **c:** X-Gal stained E11.5 wildtype *Dct::lacZ* embryo showing an under-representation of melanoblasts in the middle of the trunk (black arrow).

d: Plot of the mean M-phase length (T_m) against density (n = 19 wildtype Videos). Pearson product-moment correlation indicates no association between M-phase length and density (r = -0.15, d.f = 17, P = 0.55). **e:** Analysis of the proportion of cells in M-phase in E14.5 time-lapse sequences (n = 19 wildtype samples). Pearson product-moment correlation indicates a significant negative association between mitosis time and density (r = -0.59, d.f = 17, P = 0.007). **f:** Melanoblast cell cycle times in $Kit^{+/+}; Nf1^{+/+}$ (n = 19), $Kit^{W\text{-}v/+}; Nf1^{+/+}$ (n = 12), $Kit^{+/+}; Nf1^{+/-}$ (n = 7) and $Kit^{+/+}; Nf1^{-/-}$ (n = 14) embryonic skin cultures. No group differs significantly from wildtype ($Kit^{+/+}, Nf1^{+/+}$) (One-way ANOVA P = 0.963). **g:** Melanoblast cell cycle times corrected for cell density in $Kit^{+/+}; Nf1^{+/+}$ (n = 20), $Kit^{W\text{-}v/+}; Nf1^{+/+}$ (n = 12), $Kit^{+/+}; Nf1^{+/-}$ (n = 7) and $Kit^{+/+}; Nf1^{-/-}$ (n = 14) *ex vivo* E14.5 skin cultures. $Kit^{Wv/+}, Nf1^{+/+}$ mice have an increased cell cycle time (One-way ANOVA P < 0.0001, TukeyHSD P < 0.0001). fl = forelimb, hl = hind limb, scale bar in c = 200 µm.

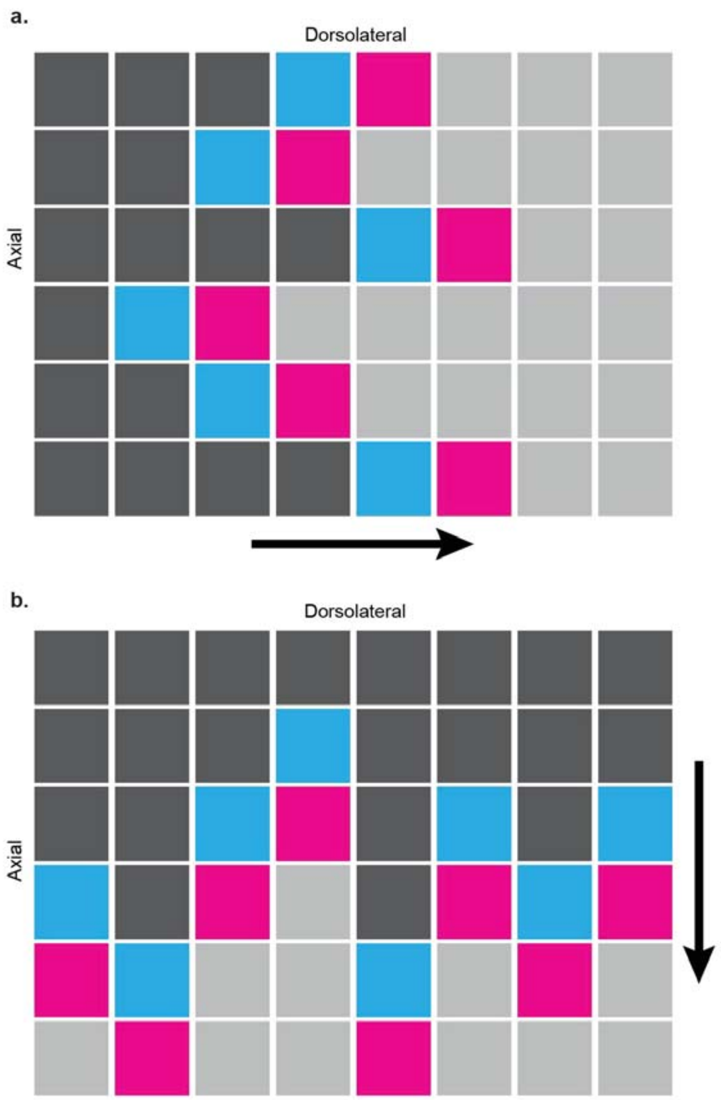

Supplementary Figure 2: Pushing growth mechanism of the discrete model. a: Growth in the horizontal direction (dorsoventral) for a two-dimensional lattice. The arrow indicates the direction of growth. For each row, a column has been chosen uniformly at random. Together the row and column index specify a site (magenta) that undergoes a growth event: the red site moves one lattice spacing to right, carrying with it any contents, and a new empty site (blue) is inserted in its place. All sites to the right of a red site's initial position move one space to the right to accommodate the new site. **b:** For growth in the axial domain the same mechanism is invoked except that cells move downwards rather than to the right after a growth event.

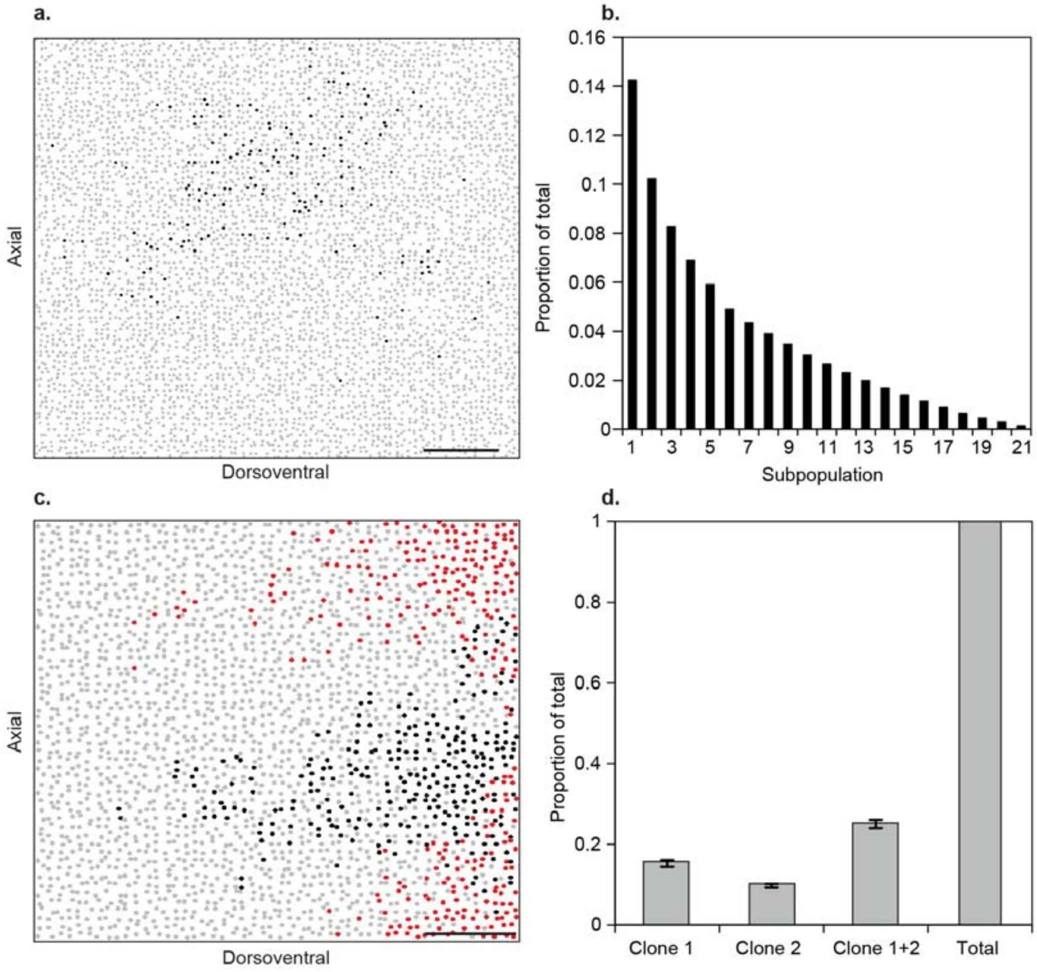

Supplementary Figure 3: Investigation of dominant sub-clones. a: To generate rare clones we chose a single agent from the entire population at a time point chosen uniformly at random and followed its progeny for the rest of the simulation, the example cropped to show the extent of a single rare clone (black cells). The clone was initialised at t = 8h 20min and plotted at t = 120hrs (the end of the simulation). **b:** Plot of the mean agent proportions for all lineages from 100 repeats of the discrete model simulation showing weak selection bias towards a smaller number of dominant lineages. **c:** A dominant lineage pattern from a single simulation of the non-growing discrete model. The two dominant lineages are shown in red and black, all other clones are in grey. **d:** Clone counts from 100 repeats of the discrete simulation without domain growth show that the top 2 dominant clones tend to contribute around 25% of the total number of cells. Error bars in d = s.e.m.

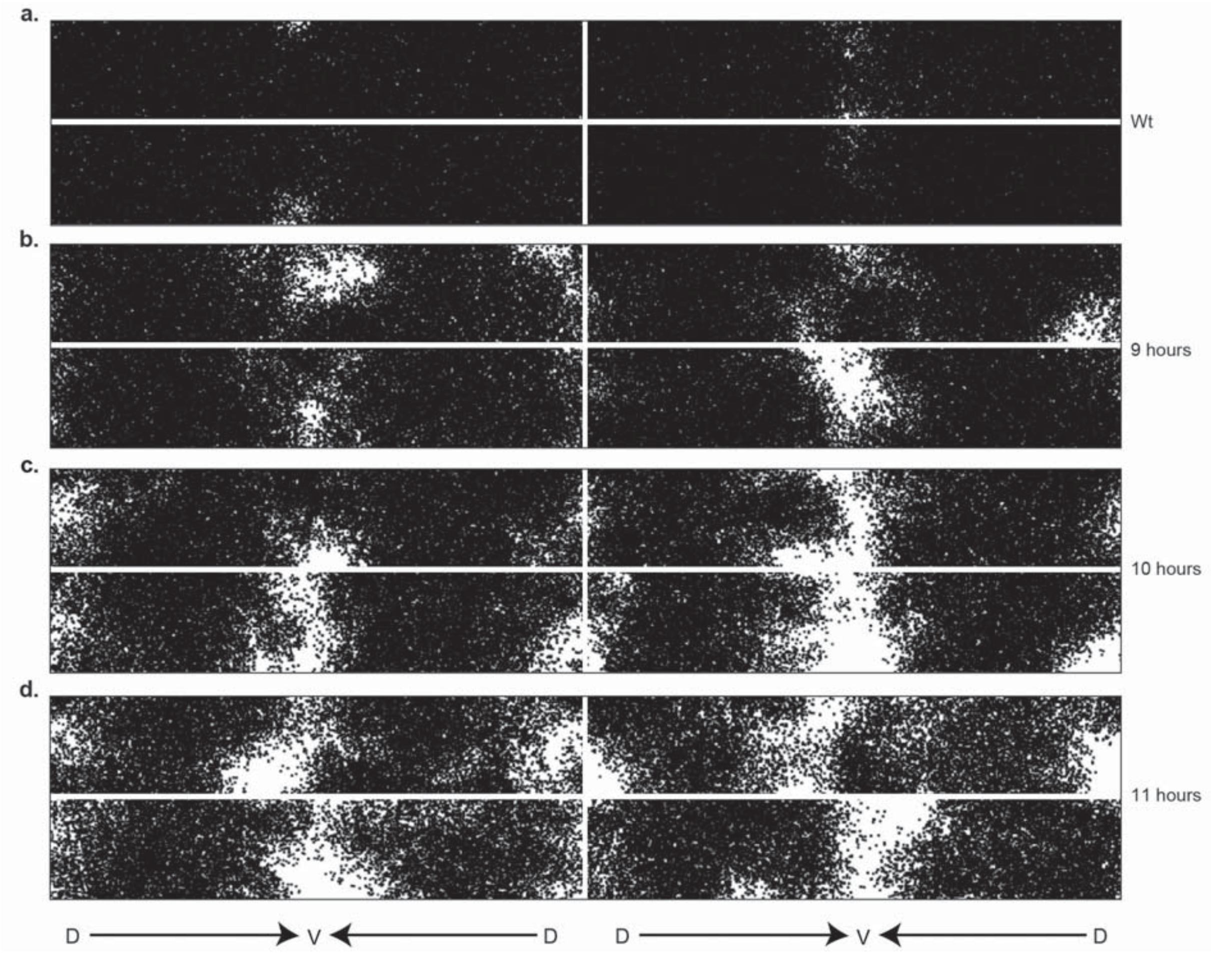

Supplementary Figure 4: Effect of increasing cell cycle time on belly spot formation. A: Four examples of left and right domains showing colonisation using the wildtype parameters (7-hour cell cycle time, the ventrum is at the centre of each panel). Some areas of slightly lower density are present at the ventrum consistent with experimental data. **b-d:** As the cell cycle time increases (from 7-11 hours) melanoblast density at the ventrum and the dorsal region decreases. In the model both ends of the domain are sensitive to proliferation the ventrum more so than the dorsum. D = dorsal, V = ventral.

Supplementary Tables

Supplementary Table 1: Analysis of the distribution of melanoblast angles

**Age**	**Melanoblast marker**	**Number of Cells**	**Kolmogorov-Smirnov *P***
E11.5	*Dct::lacZ*	325	0.2955
E12.5	*Dct::lacZ*	232	0.2817
E13.5	*Dct::lacZ*	331	0.2717
E14.5	*Tyr::Cre/R26R-EYFP*	414 independent samples	> 0.25 in all cases
E15.5	*Dct::lacZ*	318	0.2925

Supplementary Table 2: Growth of the mouse trunk between E10.5 and E15.5

**Age**	**N**	**Mean axial width (µm ± 95% CI)**	**Mean dorsoventral length (µm ±95 % CI)**
E10.5	6	1641.4 ± 160.3	1496.9 ± 95.4
E11.5	6	1845.7 ± 146.1	2461.8 ± 248.0
E12.5	9	2196.1 ± 128.1	4684.8 ± 116.2
E13.5	7	2322.5 ± 90.2	5409.1 ± 168.0
E14.5	6	2701.4 ± 172.7	6590.6 ± 205.4
E15.5	6	3080.9 ± 190.2	7901.8 ± 449.0

Supplementary Table 3: Parameters used in the discrete model

**Parameter**	**Value**
P_{ga}	0.00526 min^{-1} (one site added to each column per growth event)
P_{gd}	0.0246 min^{-1} (one site added to each row per growth event)
P_m	0.0412 min^{-1}
P_p	0.00165 min^{-1}
Site length, Δ	38 µm
Initial axial length	≈ 1634 µm
Initial dorsoventral length	≈ 1178 µm
Initial agents	21

Agent movement events occur in the model with rate P_m per unit time. Agent proliferation events occur in the model with rate P_p per unit time. The insertion of new lattice sites into the domain occurs with rates P_{ga} and P_{gd} per unit time, for growth in the axial and dorsoventral direction, respectively.

Supplementary References

1. Wilkie, A. L., Jordan, S. A. & Jackson, I. J. Neural crest progenitors of the melanocyte lineage: coat colour patterns revisited. *Development* **129,** 3349–3357 (2002).
